# Evaluating effectiveness and cost-effectiveness of a group psychological intervention using cognitive behavioural strategies for women with common mental disorders in conflict-affected rural Pakistan: study protocol for a randomised controlled trial

**DOI:** 10.1186/s13063-017-1905-8

**Published:** 2017-04-26

**Authors:** Anna Chiumento, Syed Usman Hamdani, Muhammad Naseem Khan, Katie Dawson, Richard A. Bryant, Marit Sijbrandij, Huma Nazir, Parveen Akhtar, Aqsa Masood, Duolao Wang, Mark van Ommeren, Atif Rahman

**Affiliations:** 1 University of Liverpool, Liverpool, UK; 2 Human Development Research Foundation, Islamabad, Pakistan; 3 Khyber Medical University, Peshawar, Pakistan; 4 University of New South Wales, Sydney, NSW Australia; 5 VU University Amsterdam, Amsterdam, The Netherlands; 6 Liverpool School of Tropical Medicine, Liverpool, UK; 7 Department of Mental Health and Substance Abuse, World Health Organisation, Geneva, Switzerland

**Keywords:** Mental health and psychosocial support, Cluster randomised controlled trial, Effectiveness, Nonspecialist health worker, Humanitarian, Group intervention, Women, Low- and middle-income countries, Mixed-methods evaluation, Cost effectiveness

## Abstract

**Background:**

The impact of humanitarian disasters upon mental health is well recognised. The evidence for psychological interventions for mental health is mounting, but few interventions have been rigorously tested in humanitarian settings. To be sustainable in humanitarian settings interventions need to be short, simple, deliverable by nonspecialists under supervision, and adopt a transdiagnostic approach where an array of mental health outcomes are addressed simultaneously. These elements have been incorporated into the newly developed WHO Problem Management Plus (PM+) Group intervention. The aim of this trial is to evaluate the locally adapted PM+ Group intervention for women in Swat, Pakistan.

**Methods:**

This PM+ Group trial is a two-arm, single-blind, cluster randomised controlled trial conducted in a community-based setting with women in rural Pakistan. PM+ is delivered in partnership with the Lady Health Worker (LHW) Programme which provides community-based health care to women in Pakistan. Thirty-four LHW clusters will be randomised in a 1:1 allocation ratio using a permuted-block randomisation method. Participants screened and found to meet the inclusion criteria will be allocated to either the PM+ intervention group (*n* = 306), or the control arm (*n* = 306). The manualised PM+ intervention involves five sessions, each lasting 3 h, and introduces four strategies applied by participants to problems that they are facing. It is delivered by local female facilitators with a minimum of 16 years of education who are provided with targeted training and supervision. The primary outcome is individual psychological distress, measured by levels of anxiety and depression on the Hospital Anxiety and Depression Scale at 20 weeks after baseline. Secondary outcomes include major depression, post-traumatic stress disorder, levels of social support, levels of functioning, and economic effectiveness. Intervention acceptability will be explored through an embedded qualitative study.

**Discussion:**

The PM+ Group trial will provide important evidence on the effectiveness of an empirically supported psychological treatment delivered by nonspecialists in a humanitarian setting. If proven effective, the qualitative component will inform strategies for PM+ Group scale-up in health systems in other humanitarian settings.

**Trial registration:**

Australian New Zealand Clinical Trials Registry, identifier: ACTRN12616000037404. Registered on 19 January 2016; WHO Protocol ID RPC705, v.4, 2 November 2015.

**Electronic supplementary material:**

The online version of this article (doi:10.1186/s13063-017-1905-8) contains supplementary material, which is available to authorized users.

## Background

Globally, common mental disorders account for a sizeable proportion of the burden of disease [1]. The mental health impacts of events including conflict and humanitarian disaster, and associated adverse and chronic stressors, such as displacement, poverty, unemployment, bereavement, and interpersonal conflict, are well recognised [2–4]. There is an established evidence base for the effectiveness of psychological treatments, such as cognitive behavioural therapy, for mental health problems related to trauma, loss, and extreme stressors [5]; however, with some exceptions [6–10], the majority of studies have been carried out in high-income settings or on specialist-led interventions [11]. It is important to establish whether similar interventions are effective when delivered by nonspecialists in humanitarian settings, offering feasible means of reaching the large numbers of people affected by conflict and other disasters.

Humanitarian emergencies frequently lead to destruction, death, disease/disorders, and disarray that can overwhelm local capacity [12]; for instance health and social systems lacking the human resources to reach those in need [13]. To address these challenges psychological interventions that are brief, simple, effective, deliverable by nonspecialists under supervision [14–16], and adopt a transdiagnostic approach to apply the same underlying principles across an array of common mental health problems without tailoring the protocol to specific diagnoses [17], should be developed and tested.

To ensure that services meet a range of needs and beneficiary groups it is important to develop not only individual but also group versions of interventions. A meta-analysis comparing 15 studies of individual and group psychological treatments found that while individual treatments outperformed group formats in the reduction of post-treatment symptoms, at 6-month follow-up both formats were equally as effective [18]. While individual treatments may offer greater confidentiality and personalised care, group formats have other potential advantages, including the therapeutic benefits of peer interaction and potential for group members to be therapeutic agents to each other [19]. They should also be able to reach larger numbers of people, thus may offer a cost-effective means of intervening.

Findings from the process evaluation of a pilot randomised controlled trial conducted in the conservative region of Peshawar, Pakistan indicate that a facility-based individual psychological treatment would not be accessible for many women [20]. One way to increase accessibility is to adopt a community-based approach [21], such as via Pakistan’s established Lady Health Worker (LHW) programme, where community health workers provide primary health care to women and children. Furthermore, as a result of conflict, conservative norms in Swat, the district that we are studying, appear to have heightened, impacting upon women’s access to health services [22]. Therefore, psychological interventions can be expected to be more accessible if they are embedded within trusted services, facilitating contact with women in need. In the case of Swat, the LHW programme offers a viable option for community-based group delivery of psychological interventions to women who may otherwise be unable to access care.

This study builds on and extends existing evidence relating to potentially scalable psychological interventions delivered in humanitarian settings by testing a newly developed WHO transdiagnostic intervention, PM+ Group, delivered by LHWs in Swat District, Pakistan [23]. Additionally, this study aims to contribute essential evidence in humanitarian settings by examining the effectiveness of PM+ Group that incorporates key evidence-based strategies and models of service delivery.

## Methods

### Objectives and hypothesis

The objectives of this cluster randomised controlled trial (cRCT) are to evaluate the effectiveness and cost-effectiveness of the locally adapted PM+ Group intervention for women in Swat, Pakistan as compared to enhanced usual care (EUC; defined below). The primary objective is to test the effectiveness of PM+ Group in reducing symptoms of psychological distress (anxiety and depression) in women in Swat. The secondary objectives are to assess improvements in levels of functioning, major depression, symptoms of post-traumatic stress disorder (PTSD), perceived social support, and economic outcomes. To complement this quantitative data, a qualitative component will explore intervention acceptability amongst stakeholders including participants, families, and PM+ Group facilitators.

The primary hypothesis is that women receiving PM+ Group will achieve lower psychological distress (depression and anxiety) scores in comparison to the EUC control group at 20 weeks. Secondary hypotheses are that:

1. Women receiving the intervention will report improved levels of functioning and social support, assessed at post-assessment at 7 weeks, and follow-up endpoint assessments at 20 weeks
2. Women receiving the intervention will incur lower days out of role and lower health costs at 20 weeks

### Design and setting

**Fig. 1:**
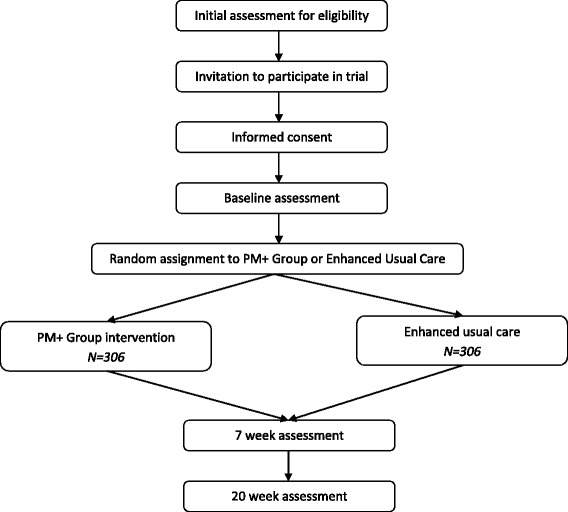
Flow chart of the study procedures

This is a two-arm, single-blind, cluster randomised controlled superiority trial conducted in a community-based setting with women in rural Pakistan. A cRCT was favoured to reduce the likelihood of contamination between the two groups. The Standard Protocol Items: Recommendations for Interventional Trials (SPIRIT) figure 2013 is provided in Fig. 1, and the SPIRIT Checklist accompanies this paper as Additional file 1.

This community-based study is being conducted in Odigram and Ghalegay Union Councils in Swat District, Pakistan. A Union Council is the smallest administrative unit in Pakistan, each with a population of approximately 25,000. Each Union Council has a Basic Health Unit (BHU) – a primary health care facility staffed by a primary care physician, a Lady Health Visitor, a vaccinator, and 15–20 community-based LHWs. LHWs are female community health workers trained to provide maternal and child health care. They receive no training in the assessment and management of common mental health problems and typically have 10 years of education. Employed by the Government, each LHW is responsible for a catchment area of approximately 1000 people or 150 homes, visiting five to seven homes daily, covering approximately 85% of the population [24, 25]. Odigram and Ghalegay Union Councils have 23 and 22 LHWs respectively. These LHW catchment areas constitute our cluster sampling frame from which 34 LHW catchment areas across the two Union Councils are involved in this study. The LHW role in supporting this study is explained below.

### PM+ Group intervention

PM+ is a brief WHO psychological intervention based on evidence of established behavioural techniques for addressing symptoms of common mental health problems in low- and middle-income countries. The manualised intervention involves empirically supported strategies of problem-solving, behavioural activation, accessing social support, and stress management training [23, 26]. Prior to implementation, a period of formative work was undertaken to contextually adapt the intervention and training materials for delivery to women in Swat, Pakistan, and a pilot trial conducted [27].

PM+ Group is an adaptation of the individual PM+ intervention. It was developed through 6 months of formative work, including review by 28 international experts. The group intervention (manual available upon request) involves five weekly sessions each lasting for 3 h, inclusive of breaks. The first session opens with psychoeducation, including information on common reactions to adversity, the rationale for PM+, goal setting, and brief motivational interviewing. Sessions 1 to 4 each introduce a strategy: (1) Managing Stress, (2) Managing Problems, (3) Get Going, Keep Doing (i.e., behavioural activation), and (4) Strengthening Social Support that are applied by participants to problems that they are facing. Each strategy is reviewed in subsequent sessions, with application of strategies between sessions encouraged to enhance learning through repetition. The final session involves a revision of learning, education on preventing relapse, and ends with a culturally appropriate closing ceremony. To enhance accessibility for illiterate individuals the programme is structured around locally relevant and appropriate pictorial materials and adopts a narrative format to support engagement and individual disclosure of personal difficulties which can be more difficult in a group format. Specifically, a case example of a woman experiencing common practical and emotional problems is shared each week, with participants following her progress through PM+ Group.

LHWs provide logistical support to PM+ Group facilitators, including the provision of a room in her house in which to conduct sessions, and convening sessions with participants from her catchment area. LHWs do not deliver the intervention, this is done by female facilitators. The PM+ Group female facilitators are recruited from Swat and have a minimum of 16 years of education and no previous experience in providing mental health care. Facilitators received 7 days of intervention training, delivered by the master trainer (KSD) and supported by three in-country supervisors (PA, HN, AM). Prior to attending training in PM+, facilitators underwent a half-day orientation to Psychological First Aid (Urdu version) [28] to sensitise them on how to appropriately respond to current crises a woman may be experiencing. Intervention training included education on adversity and its impact upon mental health, basic counselling skills, delivering PM+, skills in group facilitation, and facilitator self-care. An additional half-day security training for working in unstable settings was also provided. To build confidence and competence following training, all facilitators delivered one practice group each at an accelerated rate (five sessions in 2 weeks), with participants living outside the trial area, under intensive supervision. After conclusion of practice groups all facilitators underwent competency assessments prior to delivery of PM+ Group to cRCT participants. In case of insufficient competency additional targeted training is provided and competency assessments readministered. A co-facilitator who occupies a support role (e.g., providing child care and supporting LHWs with logistical arrangements) supports facilitators in each group. Co-facilitators participated in a half-day orientation to their role delivered by the facilitators.

Consistent with an apprenticeship model [16], protocol adherence is ensured through weekly supervision of the facilitators provided by three trained PM+ Group supervisors based in Islamabad; and fortnightly supervision of the supervisors by the master trainer. Involving all facilitators in a group, supervision lasts 2 to 3 h, conducted via Skype. Supervision entails reviewing the progress of groups including case management of participants and additional refresher training on intervention components and group facilitation skills through role play. The supervisors are in turn provided with supervision by the master trainer, conducted fortnightly via Skype and also lasting 2 to 3 h. Intervention fidelity is monitored through independent observations of 15% of randomly selected sessions of each facilitator against tailored checklists. These fidelity checks are conducted by persons trained in PM+ Group but not acting as study facilitators or supervisors.

### Enhanced usual care (EUC)

Control and intervention arm participants will continue to receive routine LHW visits on an individual basis. The care that they receive is considered to be EUC as usual care for common mental disorders in primary care in Pakistan typically involves no or placebo care leaving the majority of cases of distress undetected and unsupported. EUC will comprise:

1. Providing LHWs and primary health care providers with training in the detection and management of mental health care needs, and referral pathways
2. Providing feedback to all participants about their assessment results
3. Providing all participants with information about the options for seeking appropriate care for distress (i.e. through their LHW, the BHU, or the tertiary health care centre)

Both arms have unrestricted access to EUC through their routine health providers. The intensity of services received in both arms will be measured at 20 weeks as part of the economic evaluation (see below).

### Participant recruitment

#### Participant inclusion criteria

Cluster eligibility requires that women are living in the catchment area of LHWs participating in the trial. Individual eligibility criteria are that participants are women aged 18–60 years who screen positive on the screening tools and do not have acute medical conditions that would preclude them from attending PM+ Group sessions. Given the group intervention format, which seeks women facing similar life experiences, an upper age limit of 60 years was deemed appropriate as the life experiences of women in the upper age cohort are considered different from those of younger women.

Inclusion criteria to be invited for trial participation are: scoring both (1) above 2 on the General Health Questionnaire for common mental disorders (GHQ-12) [29, 30] and (2) above 16 on the WHO Disability Assessment Schedule for functional impairment (WHODAS) [31]. The GHQ-12 is a screening tool for identifying minor mental disorders in the general population, and consists of 12 questions scored on a 4-point Likert scale. When used for screening the GHQ-12 is scored bi-modally (i.e. 0–0–1–1), with a score range of 0–12. In previous studies in Pakistan a cut-off of 2 or higher has been reported to indicate caseness of mental disorder [29, 32, 33]. The WHODAS is a generic instrument assessing health and disability. It is applicable across all diseases, including mental disorders; and can be used with adult populations across cultures. WHODAS assesses difficulties that people are facing across six domains of functioning (cognition, mobility, self-care, getting along, life activities, and participation) during the last 30 days due to their illness. Difficulties are scored on a 5-point Likert scale of: none, mild, moderate, severe, or extreme. In this study the 12-item interviewer administered version will be used. Both measures will be used to only include those women experiencing psychological distress and impaired functioning.

Following screening, women will be verbally informed that assessment results indicate they are eligible for the trial. They will be informed about the study and invited to complete a more comprehensive baseline assessment. For women who do not meet the eligibility criteria, their results and reasons for study ineligibility will be verbally explained to them.

#### Participant exclusion criteria

Women will be assessed for the following exclusion criteria: (1) imminent risk of suicide as defined in the mhGAP intervention guide [34], (2) a severe mental disorder (e.g. psychosis, drug or alcohol dependence), or (3) severe cognitive impairment (e.g. developmental disability or dementia) as assessed by the research team. Those meeting any of these criteria will be excluded and referred to the local Psychiatry Department in Saidu Teaching Hospital, Swat, or to BHUs, depending upon their needs. Additionally, women meeting the inclusion criteria are asked if they plan on moving out of the area during the study period (3 months); such women are excluded due to unavailability for follow-up.

#### Procedure to identify eligible participants

Women will be identified using a procedure that involves LHWs developing a list of households in her catchment area. Using a computer-generated random numbers list the research coordinator selects the household screening order for the LHW to orally inform the head of each household about the study and seek agreement for the research team to attend and conduct screening. This step addresses community suspicion and conservative community norms that prevent researchers attending homes uninvited.

If in agreement, the LHW will make a list of all women aged 18–60 years in identified households, to which the research coordinator will apply a computer-generated random numbers list to determine the order for the research team to screen eligible women in each household. Two attempts will be made to reach each woman before moving to the next on the list. Once a woman has screened positive, consented to participate in the trial, and completed baseline assessments no further screening will take place in that household. Should a woman screen positive and meet the eligibility criteria but decline to take part in the research then screening of other women in that household will continue. This ensures that only one woman from each household participates in the study to protect the confidentiality of problems revealed in PM+ Group sessions.

#### Informed consent

Informed consent follows a two-step procedure which seeks to respect conservative norms whereby family permission is required for women to participate in research, as well as to mitigate against perceived hospitality norms that may motivate agreement to participate. First, potentially eligible women will be approached by the research team for written informed consent for screening. If the woman screens positive she will be invited to participate and provided with full trial informed consent. Written informed consent will be obtained at baseline assessments, which will take place at least 24 h after the Information Sheet and Consent Form have been shared and discussed with participants. Reasons for refusal to participate at either stage will be documented. At the 7-week and 20-week assessments researchers will verbally reaffirm consent with participants. For the qualitative component separate written informed consent will be taken at the time of interview.

### Sample size and power calculations

Similar community-based intervention studies using change in symptom-based questionnaires, like the Hospital Anxiety and Depression Scale (HADS) [35–37], have used effect sizes of at least 0.4 when testing treatment as usual groups with limited or no active therapeutic elements, as we propose. Assuming an effect size of 0.4 for the primary endpoint (HADS total score at 20 weeks), with 90% power and 5% significance, an intracluster correlation coefficient of 0.05, and a two-sided hypothesis test with 34 health worker catchment area clusters randomised at a 1:1 allocation ratio, and accounting for 20% attrition, we will need 612 participants (i.e. 306 in each arm). This means that we will need on average 18 participants from each of the 34 health worker catchment areas. A pilot study conducted a nearby Union Council found that these recruitment numbers were feasible and achievable [27], and previous research in the same setting identified a high prevalence of mental health problems in women [38].

### Randomisation

**Fig. 2:**
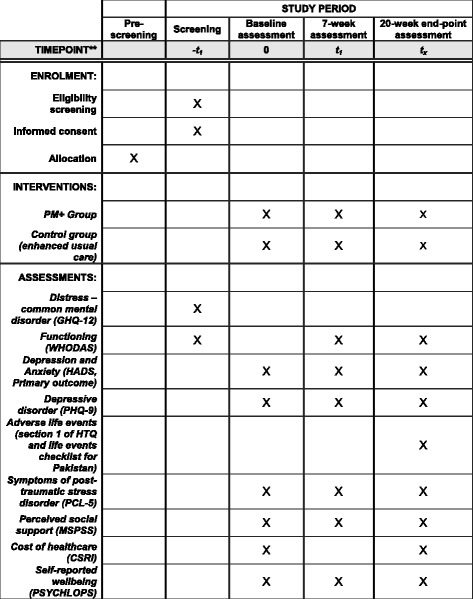
Schedule of enrolment, interventions, and assessments

Thirty-four LHW clusters will be randomised to the intervention and control arms on a 1:1 allocation ratio using a permuted-block randomisation method (see Fig. 2). A randomisation list will be generated by an independent statistician using SAS PROC PLAN. Allocation of clusters will be carried out by an independent person based at the Human Development Research Foundation, Islamabad. This is achieved by the research team sharing a list of LHWs each with a corresponding number. Allocation status will be communicated to the research team coordinator who will inform respective LHWs after consent for trial participation is obtained from participants by researchers.

If a catchment area is allocated to the intervention arm, the LHW contacts all participants within her cluster to schedule an initial meeting to introduce the participant to the intervention facilitator. This is a necessary engagement step in this setting where service uptake is dependent upon a trusted relationship with the service provider. If allocated to the control arm the LHW continues routine visits to women in her catchment area.

### Outcome measures

#### Primary outcome

The primary outcome is levels of individual psychological distress, measured by levels of anxiety and depression on the HADS [35] at 20 weeks. This 14-item scale consists of two subscales: HADS-A comprising seven items measuring anxiety, with a score range of 0–21; and HADS-D comprising seven items measuring depression, with a score range of 0–21. Higher scores indicate higher levels of anxiety and/or depression. The Urdu version of the HADS has shown acceptable validity and reliability [39].

#### Secondary outcome measures

Secondary outcomes include depressive disorder measured by the Primary Health Questionnaire [40–42]; post-traumatic stress disorder (PTSD) symptoms measured by the Post-traumatic stress disorder Check List (PCL-5) [43]; levels of social support measured by the Multi-dimensional Scale of Perceived Social Support (MSPSS) [44]; and levels of functioning as assessed by WHODAS [31]. Costs of health care are measured using the Client Service Receipt Inventory (CSRI) [45]. Self-report wellbeing outcomes are assessed using the Psychological Outcomes Profile Instrument (PSYCHLOPS) [46]. Table 1 indicates the time points for administering each instrument.

**Table 1:** Assessment instruments and administered time point

Concept (instrument)	Screening	Baseline assessments	7-week assessment	20-week assessment
Depression and anxiety (Primary outcome)		HADS	HADS	HADS
Functioning	WHODAS		WHODAS	WHODAS
Distress – common mental disorder	GHQ-12			
Depressive disorder		PHQ-9	PHQ-9	PHQ-9
Adverse life events				Section 1 of HTQ Life Events Checklist for Pakistan
Symptoms of post-traumatic Stress Disorder		PCL-5	PCL-5	PCL-5
Perceived social support		MSPSS	MSPSS	MSPSS
Cost of health care		CSRI		CSRI
Self-reported wellbeing		PSYCHLOPS	PSYCHLOPS	PSYCHLOPS

*CSRI* Client Service Receipt Inventory, *GHQ* General Health Questionnaire for common mental disorders, *HADS* Hospital Anxiety and Depression Scale, *HTQ* Harvard Trauma Questionnaire, *MSPSS* Multi-dimensional Scale of Perceived Social Support, *PCL* Post-Traumatic Stress Disorder Check List, *PHQ* Patient Health Questionnaire-9, *PSYCHLOPS* Psychological Outcomes Profile Instrument, *WHODAS* WHO Disability Assessment Schedule for functional impairment

Briefly, the Patient Health Questionnaire-9 (PHQ) is a 9-item instrument measuring the likely presence and severity of depressive disorder against the *Diagnostic and Statistical Manual of Mental Disorders, fourth edition* (DMS-IV) [42]. It uses a 4-point Likert scale where symptom severity is rated over the last 2 weeks from not having the symptom at all, to having it nearly every day. The sum of items gives the total score, with a cut-off of ≥10 as the most accurate for detecting depression [47]. The PHQ has been validated in Urdu [40, 41].

The PCL-5 is a 20-item checklist corresponding to the 20 DMS V PTSD symptoms, and has been previously used in Pakistan [48]. Items are rated on a scale of 0–4, with a total severity score of 80. The PCL-5 has been adapted to ask for symptoms in the last week, rather than the last month, to enhance sensitivity to change.

The MSPSS is a self-rating tool of perceived social support containing 12 questions rated on a 7-point Likert scale, with higher scores indicating higher levels of social support [44]. Questions correspond to three categories of support: from family, friends, and significant other. It has been translated into Urdu and validated with a Pakistani population [49].

The CSRI will be used to collect data on service utilisation and characteristics of people experiencing distress as the basis for calculating the costs of care for health [45]. It has been translated and adapted for use in Pakistan.

PSYCHLOPS is a self-report measure consisting of four questions across three domains: problems (two questions), function (one question), and wellbeing (one question). Responses are scored by domain on an ordinal 6-point scale with a maximum score of 20. The during-therapy and post-therapy versions of PSYCHLOPS consist of the same four questions, with the post-therapy version containing an additional overall valuation question determining self-rated outcome ranging from ‘much better’ to ‘much worse’. PSYCHLOPS has been validated in primary care populations across several countries [50, 51].

#### Other measures

Also assessed at 20 weeks are adverse life events measured using two instruments: firstly, Section 1 of the Harvard Trauma Questionnaire (HTQ) which includes 17 items describing traumatic events [52], validated in Pakistan [53]. In addition, day-to-day life events will be assessed using a life-events measure developed for women in Pakistan [36]. Both the HTQ and life-events measure contain additional questions about whether events have occurred since trial enrolment to enhance sensitivity.

#### Further data

At baseline we will also collect demographic data on each participant, including age, marital status, years of schooling, and occupation of the woman. Where applicable we will also collect data on number of children and their ages, and years of schooling and occupation of the woman’s husband. Research assistants also subjectively assess the socioeconomic status of the household using a locally developed rating scale.

#### Qualitative evaluation

Semistructured interviews will be conducted with a random subsample of intervention facilitators; LHWs; intervention participants with an equal number of completers and dropouts; control arm participants; senior staff with policy implementation responsibilities/connected to the research (i.e. receiving referrals of excluded study participants); research assistants; and family members of intervention participants with an equal number of intervention completers and dropouts. We anticipate up to six interviews with each category of respondent, with sampling determined by saturation. Interviews follow a semistructured topic guide that address topics relevant to each category of respondent (see Table 2).

**Table 2:** Qualitative evaluation

Category of respondent	Topics to be explored
Intervention facilitators	Overall impressions of PM+ Group Experiences of PM+ Group training and supervision Rapport with participants and families of participants Views on the group delivery format Experiences of participants’ intervention adherence and strategies to keep participants motivated View on intervention scalability
LHWs	Overall impressions of PM+ Group Strategies to keep participants motivated to attend the groups and experiences of participants intervention adherence View on intervention scalability with LHW as entry point into primary healthcare
Intervention participants – completers	Overall impressions of PM+ Group Rapport with group facilitators Intervention adherence Burden of research interviews
Intervention participants – drop outs	Reasons for stopping attending PM+ Group If appropriate: Rapport with group facilitator Intervention acceptability Burden of research
Control arm participants	Experiences of research, including participants and family views of the research assistants and research procedures Views of the questions researchers asked
Senior staff with policy implementation roles/connected to the research	Overall understanding and impressions of PM+ Group Existing scope of work of primary health care system in Pakistan, with a focus on LHW role Views on possible routes for integration and scale-up of PM+ Group into Pakistan primary health care systems
Research assistants	Views of assessment training and supervision Experiences of conducting research assessments, including the appropriateness of individual instruments, problems relating to masking, and experiences of participant distress
Family members of participants	Overall understanding and impressions of PM+ Group Where appropriate: Views of the impact of PM+ Group upon the participant in the family Views of the research assistants and assessments

*LHW* Lady Health Worker

Qualitative interviews and data analysis will be conducted by a pair of researchers independent of the research and intervention teams to avoid biasing responses. Researchers will be trained in the key principles of qualitative interviewing and also provided with supervision throughout data collection. Due to local community suspicions it is not possible to audio-record interviews; therefore, all interviews will be recorded in a written verbatim transcript produced as the interview is conducted [54]. Analysis will be conducted manually following an established thematic approach [54, 55].

### Masking

Due to the nature of the intervention it is not possible to mask participants, LHWs, and intervention facilitators and co-facilitators to treatment allocation. All researchers conducting quantitative outcome assessments will be masked, while the qualitative research team will be unmasked. Researchers conducting assessments are residents of the local Swat District and remain distinct to the intervention facilitation and co-facilitator teams with separate offices, logistical arrangements, and administrative management. They are trained and supervised by the site principal investigator (PI; NK).

Prior to each assessment point participants are instructed by LHWs not to disclose to researchers their allocation status. The researcher completes a form to guess which arm of the trial the participant belongs to both preceding and following the conduct of each assessment. The order of assessments ensures that the primary outcome measure (HADS) is administered first to minimise the risk of bias should masking be compromised. If unmasking does occur this will be documented, the assessment halted, and a new researcher assigned to complete assessments with that participant. The CSRI is administered as the final instrument at the 20-week assessment to minimise the impact upon assessments should responses lead to unmasking.

The trial statistician (DW) is blinded to the treatment code when developing the statistical analysis plan and writing the statistical programmes, which will be validated and completed using dummy randomisation codes. The actual allocation will only be provided after locking of the database.

### Data management

Quantitative data will be completed on paper assessment booklets with assigned participant codes, stored at the research field office at the end of each day. Daily checking of data will be performed by the research coordinator, with queries identified and resolved promptly. Double data entry will be conducted by an assigned data entry team at the Human Development Research Foundation (HDRF) in Islamabad, with discrepancies resolved by a third data entry person. Once in an electronic file, all data will be password-protected, with data managers controlling access to the passwords and the database backed up daily.

Qualitative data will be stored in paper format in locked filing cabinets in the research field office at the end of each day. None of the qualitative data will contain identifiers (name, age, category of respondent, etc.) that may compromise participant anonymity.

All other process data (i.e. supervision forms) will be stored in locked filing cabinets in the intervention field office. Intervention team members have been trained in the importance of de-identifying all notes relating to participant progress through the intervention to ensure confidentiality.

### Statistical methods

Data will be analysed using SAS 9.3 and SPSS Version 21. Findings will be reported according to the Consolidated Standards of Reporting Trials (CONSORT) guidelines for cRCTs [56]. Primary analyses will be based on the intention-to-treat population and secondary analyses will be based on the per-protocol population. A linear mixed model will be employed for the primary endpoint analysis. The mixed model will have treatment, visit, interaction between treatment and visit as fixed effects, baseline measurement of HADS as covariate, and cluster and subject as random effects. The mean difference between two treatment arms at each visit, together with its 95% confidence interval (CI), will be derived from the mixed model. Covariate-adjusted mixed model of primary endpoint will also be performed by adding prespecified covariates at baseline into the above model. Missing data will be treated as missing at random in the mixed model analysis and no imputation of primary endpoint will be made. To assess the sensitivity of the result to this assumption, the last observation carried forward strategy will be used to compute missing primary endpoints. Subgroup analysis will be performed on the prespecified covariates.

Continuous secondary outcomes will be analysed in a similar way as the primary endpoint analysis. For the analysis of binary secondary outcomes, a generalised mixed model will be employed with treatment, visit, interaction between treatment and visit as fixed effects, baseline measurement as covariate, and cluster and subject as random effect. The odds ratio between two treatment arms at each visit together with its 95% CI will be derived from the generalised mixed model.

All analyses will be described in detail in the finalised and signed statistical analysis plan before unmasking the study.

#### Economic analysis

Health economic analysis will be conducted to determine the difference in costs and outcomes in the intervention arm as compared to the control arm.

We take a broad public health and societal perspective to assessing economic implications, including all direct cost of health, social, voluntary and private sector services accessed by participants; productivity losses of the participant and caregivers; and informal care and out-of-pocket expenses.

Primary analysis will be of total costs over the 20-week follow-up treatment period. Recognising that cost data are often skewed, the bootstrap technique will be applied. The sampling with replacement from the original observed paired of costs and effects will be employed to maintain the correlation structure between costs and benefits, and bootstrapping sampling will be repeated 1000 times. For each bootstrap sample, an estimate of differential total mean costs and expected mean effectiveness will be calculated. The 95% CIs for the differential estimates will be derived from the 2.5th and 97.5th percentiles [57, 58].

Cost-effectiveness will be assessed by combining costs with the outcome measures including (1) HADS-A and HADS-D scores and (2) WHODAS total score in incremental cost-effectiveness analysis. Repeat resampling from the costs and effectiveness data (bootstrapping) will be used to calculate the probability that each of the treatments is the optimal choice, subject to a range of possible maximum values (ceiling ratio) that a decision-maker might be willing to pay for a unit improvement in HADS-A and HADS-D scores, and WHODAS scores.

The results of the cost-effectiveness will be reported as incremental cost-effectiveness ratios, and acceptability curves which summarise the information contained in a cost-effectiveness plane [59].

### Ethical considerations

The trial protocol has received ethical approval from the Institute of Psychiatry, Benazir Bhutto Hospital, Rawalpindi, Pakistan and the World Health Organisation Ethical Review Board. The study has also received support from the Swat District Health Department and People’s Primary Healthcare, and from the Psychiatry Department at Saidu Teaching Hospital, Swat.

#### Trial management

Overall trial management is provided by a Project Steering Committee comprised of all senior research and intervention staff, local and international PIs, technical experts, and external advisors who meet fortnightly during trial recruitment and follow-up, and monthly following the completion of endpoint assessments. The Project Steering Committee receives reports from the Trial Management Committee comprised of local research and intervention teams responsible for day-to-day trial conduct. A Trial Advisory Board comprised of persons independent of the trial with expertise in trial management and local culture meets monthly. This board is chiefly responsible for reviewing all adverse reactions and serious adverse events (SAEs) to determine if they are attributable to the trial.

#### Adverse event monitoring

All adverse reactions and SAEs reported spontaneously by the participant, or observed by research or intervention staff, will be recorded. An event is considered a potential SAE if it is an undesirable experience occurring to a participant during the study, whether or not considered related to the research procedure. The chair or a nominated person from the Trial Advisory Board will review SAEs within 48 h, deciding if it is likely related or unrelated to the intervention; and the Trial Advisory Board will review all adverse reactions twice a month. In both instances, the Trial Advisory Board will determine if any appropriate action in respect of ongoing trial conduct is necessary and specify what action this would be (i.e. referral to specialised care). The site PI will inform trial participants and those bodies providing ethical oversight if anything occurs on the basis of which it appears that the disadvantages of participation may be significantly greater than was foreseen.

## Discussion

The PM+ Group trial will provide evidence on the effectiveness of an empirically supported group psychological intervention delivered by nonspecialists in rural Pakistan, complemented by qualitative data on intervention acceptability. While various components of PM+ strategies have been proven effective [23], the combination of these strategies into a brief (five sessions) transdiagnostic structured group intervention delivered by nonspecialists in humanitarian settings has not been examined before. Critically, this study builds upon results of an individual PM+ trial [20], addressing access barriers for women by adapting the intervention to a community-based group format delivered in partnership with LHWs who are trusted and embedded community health professionals. If proven effective, WHO will make the intervention freely available on its frequently accessed website. The potential benefits of providing multiple intervention formats for reaching populations in need in a scalable and sustainable manner, addressing a range of needs in a cost-effective way, have made the case for this study all the more compelling.

### Trial status

Trial recruitment commenced December 2015 and, at the time of manuscript submission, was ongoing. Results of this study are expected in 2017.

## Additional file

Additional file 1: SPIRIT 2013 Checklist: recommended items to address in a clinical trial protocol and related documents. (DOCX 51 kb)

## Abbreviations

BHU: Basic Health Unit
cRCT: Cluster randomised controlled trial
CSRI: Client Service Receipt Inventory
EUC: Enhanced Usual Care
GHQ: General Health Questionnaire
HADS: Hospital Anxiety and Depression Scale
HDRF: Human Development Research Foundation
HTQ: Harvard Trauma Questionnaire
LHW: Lady Health Worker
MSPSS: Multi-dimensional Scale of Perceived Social Support
PCL: Post-Traumatic Stress Disorder Check List
PHQ: Patient Health Questionnaire
PM+: Problem Management Plus
PSYCHLOPS: Psychological Outcomes Profile Instrument
PTSD: Post-traumatic stress disorder
SAE: Serious adverse events
WHODAS: World Health Organisation Disability Assessment Schedule
